# The embryonic lethal mutation *zyg-10(b261)* is an allele of the *atx-2* gene and disrupts multiple aspects of early embryogenesis

**DOI:** 10.17912/micropub.biology.001714

**Published:** 2025-08-28

**Authors:** Zachary G. Bell, Harold E. Smith, Kevin F. O'Connell

**Affiliations:** 1 Laboratory of Biochemistry and Genetics, National Institute of Diabetes and Digestive and Kidney Diseases, Bethesda, Maryland, United States; 2 Genomics Core, National Institute of Diabetes and Digestive and Kidney Diseases, Bethesda, Maryland, United States

## Abstract

The *zyg-10 ( b261 )* mutation was identified in one of the earliest screens for temperature-sensitive embryonic lethal mutations in *C. elegans* , but the cytological defects underlying the embryonic lethal phenotype, as well as the molecular identity of *zyg-10* had not been previously established. Here we show that *zyg-10 ( b261 )* is an allele of the *atx-2* (ataxin-related) gene and that embryos produced by *atx-2 ( b261 )* mothers exhibit a variety of defects including eggshell defects, cytokinesis failure, spindle mispositioning, and chromosome missegregation. We also show that the localization of separase, a regulator of egg-shell formation and mitosis, is defective in *atx-2 ( b261 )* embryos.

**Figure 1. The zyg-10(b261) mutation is an allele of atx-2:**
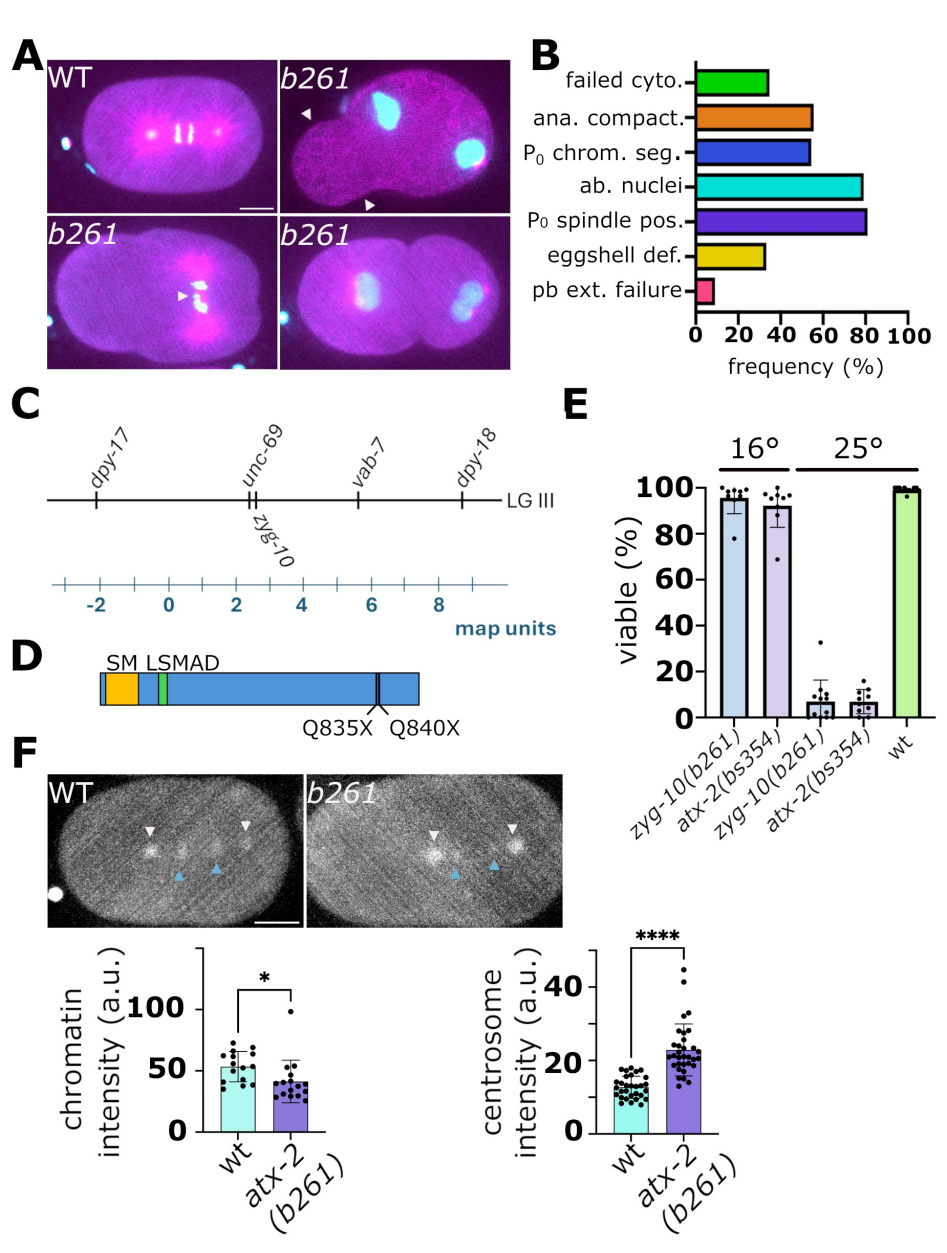
**A.** Select frames from recordings of wild-type and *zyg-10(b261)* embryos expressing GFP::histone (cyan) and mCherry::β-tubulin (magenta). At upper left is a wild-type embryo in anaphase. At upper right is a *zyg-10(b261)* embryo with a large cytoplasmic extension (arrowheads) which later resorbs, leading to a relatively normal-shaped embryo. This phenotype is likely due to an eggshell defect. At lower left is a mutant embryo with a mispositioned (transverse) spindle and lagging chromosomes (arrowhead). At lower right is a two-cell mutant embryo with paired nuclei. Scale bar is 10 μm. **B.** Quantification of defects in *zyg-1(b261)* embryos (n=24). Defects listed are failed cytokinesis (failed cyto.), loss of anaphase compaction (ana compact.), a defect where individual chromosomes disperse during anaphase often leading to multiple nuclei, P _0_ chromosome segregation defects (P _0_ chrom. seg.), which includes failure to align all chromosomes on metaphase plate and/or lagging chromosomes, abnormal nuclei (ab. nuclei) which includes mishappen or paired nuclei, P _0_ spindle mispositioning (P _0_ spindle pos.), eggshell defects (eggshell def.), and polar body extrusion failure (pb ext. failure). By comparison, 14 wild-type embryos were scored with the only defect being one instance of a failed cytokinesis. No other abnormalities were detected. **C.** Map position of *zyg-10* as determined by three factor mapping. **D.** Schematic of the ATX-2 protein showing conserved SM and LSMAD domains and positions of truncations in *atx-2(ne4297* [Q835X]) and *atx-2(b261* [Q840X]) mutants. **E.** Embryonic viability of *zyg-10(b261)* and CRISPR allele *atx-2(bs354)* at 16° and 25°. Each datapoint represents the viability of offspring from a single hermaphrodite. Wild-type is included as a control. **F.** Representative images of wild-type and *atx-2(b261)* embryos expressing endogenously tagged SEP-1::GFP. Anaphase chromatin is marked with blue arrowheads and centrosomes with white arrowheads. The position of chromatin was followed by monitoring co-expressed mCherry::histone. Scale bar is 10 μm. The graphs shown below the images plot the integrated fluorescence intensity of SEP-1::GFP on chromosomes and centrosomes. Each data point represents the intensity from all chromatin in a single embryo or of individual centrosomes. * p< 0.05, **** p<0.0001. Statistical analysis of SEP-1::GFP intensity between wt and *zyg-10(b261)* was done with an unpaired t-test using the software Prism™ 10.

## Description

The *zyg-10 ( b261 )* mutation was identified in a pioneering screen for temperature-sensitive (ts) embryonic lethal mutations in 1980 (Wood *et al.* 1980), yet the underlying cause of the embryonic lethality as well as the molecular identity of *zyg-10* has remained a mystery. To address these issues, we sought to characterize the early divisions of *zyg-10 ( b261 )* embryos expressing GFP::histone and mCherry::β-tubulin. We found that such mutants exhibit a variety of defects —most notably, a mispositioning of the P _0_ spindle, which often assembles on a transverse axis (vs longitudinal in the wild type) at the posterior of the embryo ( Figure 1A and B). This defect is consistent with earlier analysis which showed that *zyg-10 ( b261 )* embryos undergo a skewed first cleavage resulting in a smaller than normal P _1_ blastomere (Wood *et al.* 1980). However, we detected many other defects including misshapen or paired nuclei, chromosome segregation errors, and cytokinesis failure ( Figure 1A and B). Thus, the *zyg-10 ( b261 )* mutation affects many aspects of the early divisions.

We mapped *zyg-10* to position 2.6 between *unc-69* and *vab-7* on chromosome 3 ( Figure 1C ). Whole genome sequencing revealed that the *zyg-10 ( b261 )* strain contained a nonsense mutation (Q840X) in the *atx-2* gene, which is located in the same genetic interval at position 2.51. Interestingly, the existing allele *atx-2 ( ne4297 )* is also a premature stop (Q835X) mutation ( Figure 1D ) that confers a ts embryonic lethal phenotype marked by many of the same cytological defects as *zyg-10 ( b261* ) (Gnazzo *et al.* 2016; Stubenvoll *et al.* 2016). We used CRISPR to recreate the Q840X mutation, designated *atx-2 ( bs354 ),* and found that it produced a ts embryonic lethal phenotype that was similar in magnitude at both 16° and 25° to that of the *zyg-10 ( b261 )* strain ( Figure 1E ). We conclude that *b261* is an allele of the *atx-2* gene and that the phenotypes specified in Figure 1E are due to disruption of ATX-2 function.

Interestingly, *atx-2* mutant embryos share many phenotypes with embryos lacking the mitotic regulator separase, including polar body extrusion failure, chromosome mis-segregation, eggshell defects, and cytokinesis failure (Siomos *et al.* 2001; Bembenek *et al.* 2007; Richie *et al.* 2011; Gnazzo *et al.* 2016; Stubenvoll *et al.* 2016). Thus, we decided to analyze the subcellular distribution of separase in *atx-2 ( b261 )* embryos. Focusing on the portion of separase at the spindle, we found that SEP-1 levels were decreased moderately but significantly on chromatin, suggesting a possible cause for some of the chromosome segregation defects ( Figure 1F ). We also found that *atx-2 ( b261 )* embryos have more separase at the spindle poles, possibly as a result of the enlarged centrosomes that are observed in the absence of ATX-2 (Stubenvoll *et al.* 2016). In summary we have established the molecular identity of *zyg-10* and provide evidence suggesting that mislocalization of separase may contribute so some of the defects observed in the absence of ATX-2 .

## Methods

For live imaging of embryos expressing GFP::histone and mCherry::β-tubulin, L4 stage worms were shifted to 25°C, 24 hours prior to imaging. Embryos were then dissected from gravid hermaphrodites in a 2 µl drop of egg buffer (188 mM NaCl, 48 mM KCl, 2 mM CaCl _2_ , 2 mM MgCl _2_ , 25 mM HEPES, pH 7.3) on a 12 mm circular coverslip. The coverslip was inverted and placed on a 3% agar/egg buffer pad that was cast in the well of a 0.25 mm thick ring-shaped spacer made of CultureWell™ Sheet Material (Grace Bio-Labs, Inc. Bend, OR) on a glass slide. A second glass slide was used to flatten the agar pad to the thickness of the spacer. Slides with embryos were transferred to a Thermo Plate heating/cooling stage (Tokai Hit USA Inc, Bala Cynwyd, PA) set at 25°C and were imaged using a spinning disk confocal microscope as previously described (Sankaralingam *et al.* 2024).

For live Imaging of SEP-1 ::GFP-expressing embryos, L4 stage worms were shifted to 24°C, 24 hours prior to imaging. Embryos were then dissected from gravid hermaphrodites in a 6 µl drop of meiosis medium (25 mM Hepes pH 7.4, 60% Leibowitz L-15 Media, 20% FBS, 500 µg/ml inulin) (Audhya et al. 2005) on a 12 mm circular coverslip. A glass slide containing a ring-shaped spacer as described above was then placed on top of the coverslip allowing the embryos to stay in suspension without pressure. Embryos were imaged and analyzed as above. All image analysis and processing was performed using ImageJ2 version 2.14.0/1.54f.

To determine the molecular identify of *zyg-10* , the *zyg-10 ( b261 )* mutation was positioned on chromosome 3 via three-factor mapping as previously described (Brenner 1974). The *zyg-1 ( b261 )* chromosome was placed over the *unc-69 ( e587 ) dpy-18 ( e364 )* chromosome or the *dpy-17 ( e164 ) vab-7 ( e1562 )* chromosome and hermaphrodites allowed to produce self-progeny. Recombinant (Dpy non-Unc/Unc non-Dpy or Dpy non-Vab/Vab non-Dpy respectively) progeny were then identified and picked to individual plates. For each recombinant, a line homozygous for the recombinant chromosome was established and the presence of the *zyg-10 ( b261 )* mutation determined by screening for embryonic lethality at restrictive temperature.

For whole genome sequencing, worms were washed off a 100 mm high growth MYOB plate (3.5 mM Tris-HCl, 2 mM Tris- OH , 34 mM NaCl, 0.02 mM cholesterol , 20 g/L peptone, 30 g/L agar ) in M9 buffer (22 mM KH _2_ PO _4_ , 22 mM Na _2_ HPO _4_ , 85 mM NaCl, 1 mM MgSO _4_ ), spun for 3 minutes at 2095 x g and the excess M9 removed. The PureLink™ Genomic DNA Mini Kit (Catalog No. K1820-00, Thermo Fisher Scientific, Inc., Waltham, USA) was used to prepare genomic DNA following the “Mammalian Tissue and Mouse/Rat Tail Lysate” protocol. DNA concentration was measured using PicoGreen® and a TBS-380 Mini-Fluorometer (Turner BioSystems, Sunnyvale, USA). 100 μg of the genomic DNA was sheered using the Covaris S220 Focused-ultrasonicator and SonoLab 7 program (Covaris, LLC, Woburn, USA) to achieve an average fragment size of 300 base-pairs. A DNA library was prepared from the sheared DNA using the NEBNext® Ultra™ II DNA Library Prep Kit for Illumina®(Catalog No. E7103S, New England Biolabs, Ipswich, USA). The concentration of each library was again measured on the Mini-Fluorometer and quality assessed using the Agilent 2100 Bioanalyzer (Agilent Technologies, Inc, Savage, USA). The library was sequenced on the illumina HiSeq 3000 instrument (Illumina, Inc, San Diego, USA). Variant analysis was performed as described previously (SMITH, 2022) using a pipeline of BBMap 38.96 (BUSHNELL, 2022), SAMtools 1.16.1 (LI et al. *,* 2009), FreeBayes 1.3.5 (GARRISON and MARTH, 2012), and ANNOVAR 2020-06-08 (WANG et al., 2010) with WormBase version WS277 as the reference genome. The sequencing data has been deposited at the Short Read Archive ( https://www.ncbi.nlm.nih.gov/sra ) under accession number SRR34981507.

CRISPR -Cas9 genome editing was performed as described previously (Sankaralingam *et al.* 2024)

## Reagents

**Table:** 

	**Genotype**	Source
DH261	*atx-2 ( b261 [Q840X]) III *	CGC
OC1334	*atx-2 ( bs354 [Q840X]) III *	OC
OC908	*bsSi30 [pCW9: unc-119 (+) p cdk-11.2 ::sfgfp:: his-58 :: cdk-11.2 3' utr] II; bsIs20[pNP99: unc-119 (+) tbb-1p::mCherry:: tbb-2 :: tbb-2 3'-utr]; bsIs2 [pCK5.5: Ppie-1::gfp:: spd-2 ] *	OC
OC1250	*bsSi30 [pCW9: unc-119 (+) p cdk-11.2 ::sfgfp:: his-58 :: cdk-11.2 3' utr] II; atx-2 ( b261 ) III; bsIs20[pNP99: unc-119 (+) tbb-1p::mCherry:: tbb-2 :: tbb-2 3'-utr] *	OC
CB2233	*unc-69 ( e587 ) dpy-18 ( e364 ) III *	CGC
WM31	*dpy-17 ( e164 ) vab-7 ( e1562 ) III *	CGC
OC1329	*sep-1 ( it214 [ sep-1 ::gfp]) I, ltIs37 [pie-1p::mCherry:: his-58 + unc-119 (+)] IV *	OC
OC1331	*sep-1 ( it214 [ sep-1 ::gfp]) I; atx-2 ( b261 ) III; ltIs37 [pie-1p::mCherry:: his-58 + unc-119 (+)] IV *	OC

**Table:** 

**Reagents**	**Source**
CultureWell™ Silicone Sheet Material	Grace Bio-Labs Cat # CWS-S-0.25
12 mm circular cover glass	Fisher Scientific Cat# 12541001
PureLink™ Genomic DNA Mini Kit	ThermoFisher Scientific Cat # K1820-00
NEBNext® Ultra™ II DNA Library Prep Kit for Illumina®	New England Biolabs, Cat # E7103S

**Table:** 

**CRISPR**	**Sequence**
b261 crRNA	5'-UCAACAGUAUAUGGUGAUGC-3'
*b261* Repair Template	5'-GCAGCAGCAGCAGCAACACATTCAACAGTATATGGTGATGtAGGGCCCGC ATCAAATGCATCCGCAGATCCCTAATTACTATCAGC-3'
